# Stereotactic body radiotherapy (SBRT) for adrenal metastases of oligometastatic or oligoprogressive tumor patients

**DOI:** 10.1186/s13014-020-1480-0

**Published:** 2020-02-04

**Authors:** Laila König, Matthias F. Häfner, Sonja Katayama, Stefan A. Koerber, Eric Tonndorf-Martini, Denise Bernhardt, Bastian von Nettelbladt, Fabian Weykamp, Philipp Hoegen, Sebastian Klüter, Matthew S. Susko, Jürgen Debus, Juliane Hörner-Rieber

**Affiliations:** 1 Department of Radiation Oncology, Heidelberg University Hospital, Im Neuenheimer Feld 400, 69120 Heidelberg, Germany; 2 Heidelberg Institute of Radiation Oncology (HIRO), Heidelberg, Germany; 3 National Center for Tumor diseases (NCT), Heidelberg, Germany; 4 Clinical Cooperation Unit Radiation Oncology, German Cancer Research Center (DKFZ), Heidelberg, Germany; 5 Heidelberg Ion-Beam Therapy Center (HIT), Department of Radiation Oncology, Heidelberg University Hospital, Heidelberg, Germany; 6 German Cancer Consortium (DKTK), partner site Heidelberg, Heidelberg, Germany; 7 Department of Radiation Oncology, University of California – San Francisco, San Francisco, USA

**Keywords:** Adrenal gland metastases, Stereotactic body radiotherapy (SBRT), Image-guided radiotherapy, Oligometastases, Oligoprogression

## Abstract

**Introduction:**

Local ablative treatment strategies are frequently offered to patients diagnosed with oligometastatic disease. Stereotactic body radiotherapy (SBRT), as ablative treatment option, is well established for lung and liver metastases, whereas for isolated adrenal gland metastases the level of evidence is scarce.

**Material and methods:**

This single-institution analysis of oligometastatic or oligoprogressive disease was limited to patients who received SBRT to adrenal metastasis between 2012 and 2019. Patient, tumor, treatment characteristics, and dosimetric parameters were analyzed for evaluation of their effect on survival outcomes.

**Results:**

During the period of review 28 patients received ablative SBRT to their adrenal gland metastases. Most common primary tumors were non-small cell lung cancers (46%) with most patients diagnosed with a single adrenal gland metastasis (61%), which occurred after a median time of 14 months. SBRT was delivered to a median biological effective dose at α/β of 10 (BED_10_) of 75 Gy (range: 58–151 Gy). Median gross tumor volume (GTV) and median planning target volume (PTV) were 42 and 111 mL, respectively. The homogeneity and conformity indices were 1.17 (range: 1.04–1.64) and 0.5 (range: 0.4.0.99), respectively, with the conformity index being affected by dose restrictions to organs at risk (OARs) in 50% of the patients. Overall response rate based on RECIST criteria was 86% (CR = 29%, PR = 57%) with 2-year local control (LC) of 84.8%, 2-year progression-free survival (PFS) of 26.3%, and 1-and 2-year overall survival (OS) of 46.6 and 32.0%, respectively. During follow up, only two local recurrences occurred. A trend for superior LC was seen if BED_10_ was ≥75Gy (*p* = 0.101) or if the PTV was < 100 ml (*p* = 0.072). SBRT was tolerated well with only mild toxicity.

**Conclusion:**

SBRT for adrenal metastases resulted in promising LC with low toxicity. Treatment response appeared to be superior, if SBRT was applied with higher BED. As the close proximity of OARs often limits the application of sufficiently high doses, further dose escalations strategies and techniques should be investigated in future.

## Introduction

The adrenal glands represent a common site for cancer metastases for a variety of primary tumors, with historic autopsy series reporting an incidence of 9–27% [1]. The majority of adrenal metastases are accounted for by primary lung tumors, however breast, kidney, stomach, hepatobiliary carcinoma, and melanoma have also been found to metastasize to the adrenal glands [2, 3]. Traditionally, local therapies like surgical resection or radiotherapy were reserved for palliation of symptomatic lesions, with external beam radiotherapy demonstrating good response rates and high rates of pain relief (40–80%) [4–6].

In the contemporary setting, local ablative treatment strategies for metastases are frequently offered for patients diagnosed in an oligometastatic or oligoprogressive tumors, irrespective of symptomatology [7–9]. Two recently published prospective phase II studies demonstrate that the addition of local ablative treatment to standard of care palliative therapy for oligometastatic tumor patients not only prolonged progression-free survival (PFS), but also overall survival (OS) [10, 11]. Additionally, some surgical series report superior outcomes following adrenalectomy in selected patients with isolated adrenal gland metastases [12–15]. However, many patients with adrenal gland metastases are considered medically inoperable due to severe comorbidities or the location or the extent of their metastases [16]. For these patients, stereotactic body radiotherapy (SBRT) offers an excellent, noninvasive treatment alternative [4, 16].

In contrast to conventionally fractionated radiotherapy, SBRT allows for the delivery of highly conformal doses or radiation, enabling tumor ablation. While SBRT is well established and widely applied in the treatment of pulmonary and hepatic oligometastases [17–20], the literature for adrenal metastases is scarce. Predominantly small and retrospective studies exist which do not primarily focus on oligometastatic or oligoprogressive disease, but also include patients treated with palliative intent [21–27]. Furthermore, these studies only report limited details on radiotherapy planning and requirement for forced dose restrictions due to the close proximity to radiosensitive organs-at risks (OARs). The aim of the current study was therefore to evaluate outcomes and potentially related dosimetric parameters in oligometastatic or oligoprogressive patients treated with SBRT for adrenal metastases.

## Materials and methods

### Patient population

This study is a retrospective, single-institution analysis of oligometastatic (1–5 metastases), or oligoprogressive patients, who were treated for adrenal metastases with SBRT between October 2012 and January 2019. Institutional databases were reviewed for demographic, pathologic, radiologic, and treatment-related information. Ethical approval was sought from the Ethical Committee of the University Hospital of Heidelberg (S-627/2019). Patients were excluded from the analysis if they were not treated with SBRT but with palliative intent or palliative doses. SBRT was defined as an ablative dose with single fraction doses > 4 Gy and number of fractions < 10. All patients were either classified medically or technically inoperable. No patient suffered from symptoms related to the adrenal gland metastasis.

### Planning and treatment features

For treatment planning, computed tomography (CT) scans with and without contrast and a slice thickness of 3 mm were acquired under shallow breathing. To account for respiratory motion the preference was to obtain a 4-dimensional (4-D) CT scan during normal breathing whenever feasible (*n* = 24 patients with 4D-CT). Contrast-enhanced abdominal magnetic tomography imaging (MRI) and fluoro-deoxy-glucose positron emission tomography (FDG-PET) imaging were further applied for treatment planning in 7 patients each. Gross tumor volumes (GTV) included the macroscopic tumor on the contrast-enhanced CT, FDG-PET-CT and MR scans, with an internal target volume (ITV) being defined based on the particular tumor motion assessed on the 4-D-CT scans. To account for microscopic infiltration, a 5 mm safety margin was applied to form the clinical target volume (CTV). An additional planning target volume (PTV) margin of 2 mm was further added to account for positioning insecurities. If no 4-D-CT data was available, a PTV margin of 10 mm was used. Total dose and fractionation schemes were at the discretion of the treating physician and dependent on the adjacent normal tissue tolerances. As adrenal SBRT performed with higher biological effective doses (BED) is associated with superior local control (LC) treatment with maximally safe doses was generally pursued [26]. Applied dose constraints were in line with published guidelines [18, 20, 28]: e.g. liver dose to ≥ 700 ml < 15 Gy (3 fractions) and < 18.5 Gy (5 fraction) < 24 Gy (10 fractions), spinal cord D_0.1cc_ < 21.5 Gy (3 fractions), <30Gy (5 fractions) < 35 Gy (10 fractions), stomach/small bowel D_0.5cc_ < 25.2 Gy (3 fractions), < 35 Gy (5 fractions) and < 43.5 Gy (10 fractions). Volumetric image guidance for patient positioning using either kV cone beam CT (CBCT) or MV CT was performed before each treatment fraction.

The biological effective dose (BED) was calculated for correlating irradiated doses with clinical results: an α/β ratio of 10 Gy was assumed for the adrenal metastases. BED was determined using the linear-quadratic model [29]:

$$ \mathbf{BED}\ \left(\mathbf{Gy}\right)=\mathbf{fractional}\ \mathbf{dose}\times \mathbf{number}\ \mathbf{of}\ \mathbf{fractions}\ \left(\mathbf{1}+\frac{\mathbf{fractional}\ \mathbf{dose}}{\boldsymbol{\upalpha} /\boldsymbol{\upbeta}}\right) $$

Further quantitative and qualitative dose evaluations were conducted with dose parameters being extracted from dose volume histograms (DVH) to asses target coverage, homogeneity and conformity of the PTV. For homogeneity index (HI), a formula was used, where D5% and D95% are the minimum doses in 5 and 95% of the target volume, respectively. The ideal HI value is 1 and increases as the plan becomes less homogeneous [30].

$$ \mathbf{Homogeneity}\ \mathbf{index}\ \left({\mathbf{HI}}_{\mathbf{5}/\mathbf{95}}\right)=\frac{{\mathbf{D}}_{\mathbf{5}\%}}{{\mathbf{D}}_{\mathbf{95}\%}} $$

The conformity index (CI) was calculated according to the RTOG guidelines [31] by division of the PTV covered by the 95% isodose (reference isodose) and the target volume itself. A value close to 1 corresponds to ideal conformity.

$$ \mathbf{Conformity}\ \mathbf{index}\ \left(\mathbf{CI}\right)=\frac{{\mathbf{PTV}}_{\mathbf{95}\%}}{\mathbf{PTV}} $$

### Outcome evaluation

All patients received follow-up visits at the Heidelberg University Hospital or the Thoraxklinik Heidelberg and underwent a clinical examination and a contrast-enhanced CT scan 6–8 weeks after the completion of treatment. This was repeated thereafter at 3- to 6-months intervals depending on the primary tumor entity and the overall performance status. Treatment-related toxicity was evaluated and classified according to CTCAE v5.0. Local control (LC) was defined as no progressive disease of the treated metastasis, with treatment response being scored using the Response Evaluation Criteria in Solid Tumors (version 1.1). Treatment response to adrenal SBRT was classified as complete response (CR), partial response (PR), stable disease (SD), or progressive disease (PD).

### Statistical analysis

The statistical analysis aimed to evaluate the efficacy of SBRT for adrenal metastases and to identify prognostic factors associated with outcomes. Survival analyses for local control (LC), progression-free survival (PFS) as well as overall survival (OS) following SBRT were performed using the Kaplan-Meier method. Univariate Cox proportional hazard models were used to assess the potential influence of all patient, tumor, and treatment characteristics on LC, PFS and OS. Multivariate analysis was not attempted due small sample size and the exploratory nature and of this analysis. A *p*-value ≤0.05 was considered statistically significant. All statistical analyses were performed using the software SPSS 24.0 (IBM Corporation, Armonk, NY, USA).

## Results

### Patient and treatment characteristics

Between October 2012 and January 2019, 56 patients were treated with radiotherapy to their adrenal gland metastasis in our department. Twenty eight patients were excluded due to treatment with palliative doses. Of the remaining 28 patients available for further analysis, the median age was 63 years (range 48–91 years). Seventy-five percent of the patients met criteria of for an oligometastatic tumor stage with 0–5 additional metastases. All additional metastases, as well as the primary tumor, received further treatment with surgery or radiotherapy with or without systemic therapy. Twenty-five percent of the patients were classified to have an oligoprogredient tumor state, meaning that all additional metastatic locations including the primary tumor had previously been controlled at the time of adrenal SBRT. Most common primary tumors were non-small cell lung cancers (46%), small cell lung cancers (18%) and renal cell carcinomas (7%). The majority of patients were diagnosed with a single adrenal gland metastasis (61%), which occurred metachronously after a median time of 14 months. Detailed patient and tumor characteristics are displayed in Table 1.

**Table 1:** Patient, tumor and treatment characteristics

	Total number of patients (%)	
Gender		
Male	21 (75.0%)	
Female	7 (25.0%)	
Median Age (range) in years	63 (48–91)	
Median KPS (range) in %	80 (60–100%)	
Primary Tumor Site		
NSCLC	13 (46.4%)	
SCLC	5 (17.9%)	
CRC	1 (3.6%)	
HCC	1 (3.6%)	
Breast cancer	1 (3.6%)	
Melanoma	1 (3.6%)	
RCC	2 (7.1%)	
Other	4 (14.3%)	
Oligometastatic	21 (75%)	
Oligoprogressive	7 (25%)	
Total no of metastatic sites		
0	17 (60.7%)	
1	2 (7.1%)	
2	2 (7.1%)	
3	1 (3.6%)	
> 5	6 (21.4%)	
Laterality		
Right	14 (50.0%)	
Left	14 (50.0%)	
Metastasis status		
Synchronous	10 (35.7%)	
Metachronous	18 (64.3%)	
Median time from primary diagnosis to adrenal gland metastasis (range) in months	14 (0–102)	
Homogeneity distribution (%)		
Yes	26 (92.9%)	
No	2 (7.1%)	
Forced dose restriction (%)		
Yes	14 (50.0%)	
No	14 (50.0%)	
RT Technique		
3D conformal	4 (14.3%)	
Helical IMRT (Tomotherapy)	13 (46.4%)	
VMAT	11 (39.3%)	
FFF photons utilized		
Yes	18 (64.3%)	
No	10 (35.7%)	
Concurrent systemic therapy		
No	19 (67.9%)	
Yes	9 (32.1%)	
Chemotherapy	6 (66.7%)	
Targeted therapy	2 (22.2%)	
Immunotherapy	1 (11.1%)	
	Mean	Median (range)
Total dose (Gy)	47.3	50 (30–54)
Fractions (n)	9	10 (3–12)
Single dose (Gy)	5.6	5 (4–18)
BED_10_ (Gy)	73.5	75 (57.6–151.2)
Prescribed isodose line (%)	89	90 (80–90)
Median GTV volume (range) in cm^3^	27	42 (3–233)
Median PTV volume (range) in cm^3^	111	96 (16–346)

*KPS* karnofsky performance score, *NSCLC* non-small cell lung cancer, *SCLC* small cell lung cancer, *CRC* colorectal cancer, *HCC* hepatocellular cancer, *RCC* renal cell carcinoma, *PTV* planning target volume, *BED*_*10*_ biologically effective dose at α/β of 10, *IMRT* intensity-modulated radiotherapy, *VMAT* volumetric modulated arc therapy, *FFF* flattening filter free

SBRT was delivered with a median dose of 50 Gy (range: 30–54) with a median number of fractions of 10 (range 3–12), corresponding to a median BED_10_ of 75 Gy (range 58–151 Gy). Doses were predominantly prescribed homogenously (93%). Nevertheless, in 50% of the patients dose restrictions were necessary to spare surrounding OARs, such as the small bowel and the stomach.

### Planning and Dosimetric characteristics

Median GTV and median PTV were 42 ml (range 3-233 ml) and 111 ml (range 16-346 ml), respectively. Target volume coverage was analyzed using D_98_ (%) and D_2_ (%) of the prescribed doses to the GTV and PTV. Median D_98_ of the GTV and the PTV was 100% (range: 70–120%) and 80% (range: 60–100%). Median D_2_ of the GTV and the PTV was found to be 100% (range: 100–130%) and 100% (range: 100–140%). To further objectify this analysis, we calculated homogeneity and conformity indices for all plans. The HI showed a median value of 1.17 (range: 1.04–1.64) and the CI a median of 0.5 (range 0.4.0.99), with the latter being effected by dose restrictions to OARs.

Delivery techniques comprised 3D conformal technique (*n* = 4), helical IMRT (intensity-modulated radiotherapy) (*n* = 13) and VMAT (volumetric modulated arc therapy) (*n* = 31). In 18 plans, flattening filter free (FFF) techniques were used.

### Treatment outcomes and toxicity

Median follow up after initial tumor diagnosis was 36.4 months (range: 7.2–161.3 months) and 9.8 months (range 3.1–83.9 months) after adrenal SBRT. Clinical response after SBRT evaluated using RECIST criteria revealed a local overall response rate (ORR) of 86% (CR = 29%, PR = 57%), illustrated in Table 2. Only two patients suffered from a local relapse, leading to a 1- and 2-year LC rate of 84.8%. The first patient suffered from a NSCLC (adenocarcinoma) and his adrenal metastasis was treated with a BED of 67.5 Gy. He relapsed locally after 8.2 months without systemic failure and died due to pneumonia and pulmonary embolism some days after diagnosis of local failure. The second patient was also diagnosed with a NSCLC (adenocarcinoma), his adrenal metastasis was treated with a BED of 67.2 Gy and he relapsed only locally after 7 months. His progressive adrenal gland metastasis was resected (R1 status). However, 6 months after this operation, he was diagnosed with systemic failure (peritoneal carcinosis) and died another month after due to systemic progression.

**Table 2:** Treatment outcomes according to RECIST

	Total number of patients (%)
Clinical response	
CR	8 (28.6%)
PR	16 (57.1%)
SD	2 (7.1%)
PD	2 (7.1%)
Distant recurrence	
Yes	19 (67.9%)
No	9 (32.1%)

*CR* complete remission, *PR* partial remission, *SD* stable disease, *PD* progressive disease

**Fig. 1:**
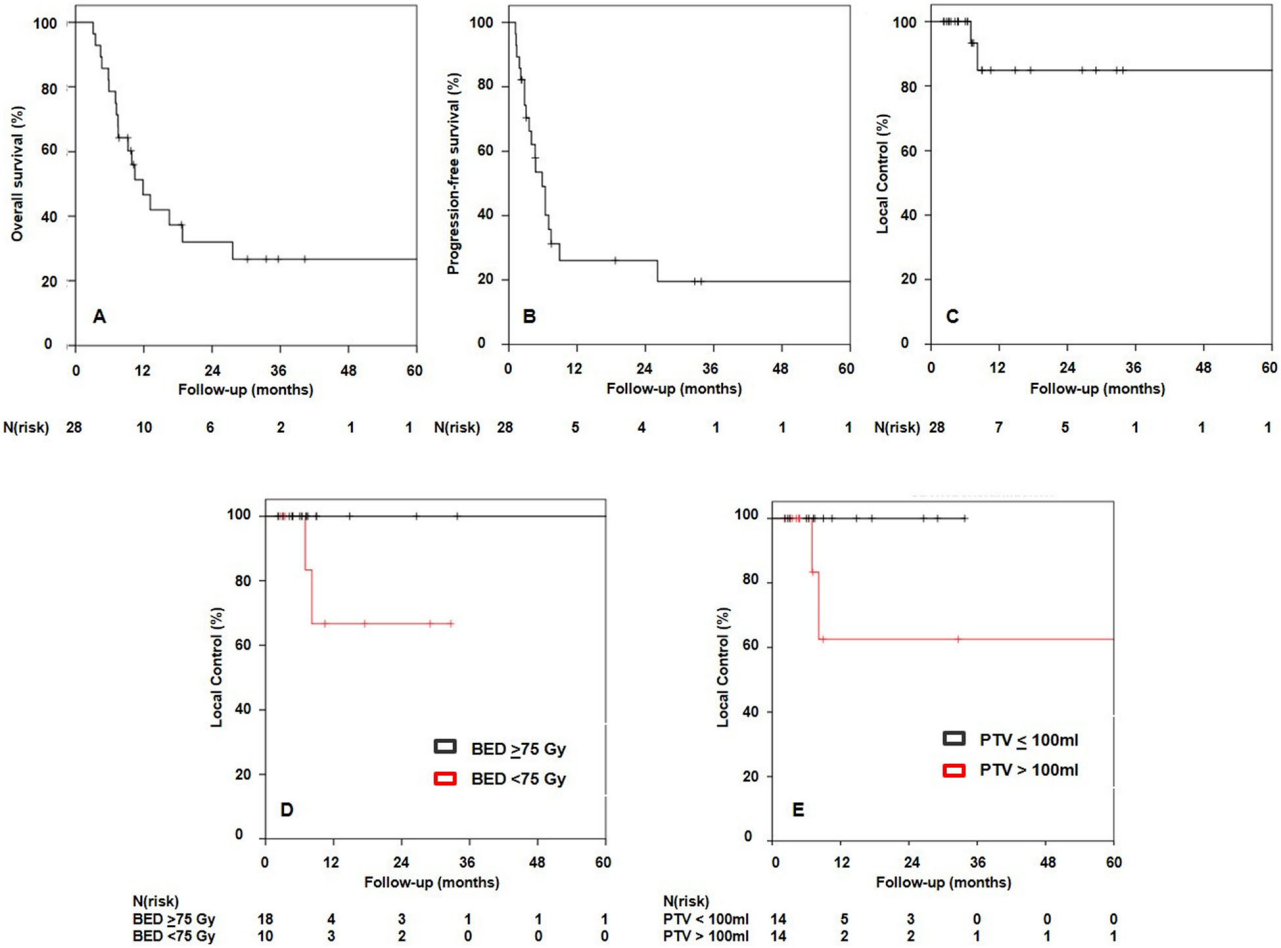
Kaplan Meier survival curves for overall survival, progression-free survival and local control

The majority of patients (68%) were diagnosed with distant relapse with a 1- and 2-year PFS rate of 26.3% (as shown on Fig. 1). During follow up, 18 patients died, resulting in a 1-and 2-year OS rate of 46.6 and 32.0%. Most the deaths (83%) were due to systemic tumor progression, while the other 17% were non-cancer-related. Median follow-up for patients who were still alive was higher with 26.1 months. Outcome data are displayed in Fig. 1. Various patient, tumor and treatment characteristics (age, sex, response to SBRT, BED, single dose, number of fractions, GTV and PTV, synchronous vs. metachronous disease, oligoprogredient vs. oligometastatic disease) were analyzed as prognostic factors for LC, PFS and OS. No significant prognostic correlations were observed. However, we detected a non-significant trend for superior LC if a BED ≥75Gy (*p* = 0.101) was applied or if PTV < 100 ml (*p* = 0.072) (see Fig. 1).

SBRT was tolerated well with only mild (grade I-II) acute toxicity in 32% of the patients. Most common acute side effects were CTCAE° I and II fatigue in 2 and 4 patients, respectively and CTCAE° I and II gastrointestinal toxicity in 2 and one patients, which were nausea (one patient needed antiemetic medication = grade 2) and loss of appetite without alteration in eating habits (grade 1). Late toxicity occurred in only 3 patients. No severe acute or chronic toxicity (>grade II) was observed during follow up. Two patients suffered from fatigue, only one patient was diagnosed with mild gastrointestinal toxicity, which was a loss of appetite without alteration in eating habits (grade 1). Detailed toxicity criteria are shown in Table 3.

**Table 3:** Toxicity according to CTCAE criteria

	Grade 1 n (%)	Grade 2 n (%)
Acute toxicity	4 (14.3%)	5 (17.9%)
Fatigue	2 (50.0%)	4 (80.0%)
Gastrointestinal	2 (50.0%)	1 (20.0%)
Abdominal pain	0	0
Anorexia	0	0
Chronic toxicity	2 (7.1%)	1 (3.2%)
Fatigue	1 (50.0%)	1 (100.0%)
Gastrointestinal	1 (50.0%)	0
Abdominal pain	0	0
Anorexia	0	0

## Discussion

With the widespread use of regularly performed staging with CT or positron-emission-tomography (PET)-CT, and the growing recognition of the oligometastatic or oligoprogressive state in different tumor entities, the number of patients presenting with asymptomatic adrenal metastases has increased [32–34]. For these patients, surgical resection remains the gold standard, however, many patients are medically unfit for surgery, or their tumors are not technically resectable, and alternative local treatment approaches are required [4, 16, 35].

As the current literature on adrenal SBRT is still limited, a patterns-of-care analysis for patients treated with hypofractionated radiotherapy to their adrenal metastases was conducted. We selected only oligometastatic or oligoprogressive patients who received high-dose SBRT to their adrenal metastases with curative intent in order to understand the role of ablative radiation in this cohort.

In our study of 28 patients, adrenal SBRT led to promising local control accompanied by only mild toxicity. In detail, we detected a 1-year and 2-year LC rate of 84.8%, which compare favorable to other studies [26, 27, 36–39]. A complete response was achieved in 8 (29%) lesions, a partial response in 16 (57%) and stability in 2 (7%) of the patients according to the RECIST criteria. Two patients were diagnosed with local relapse at 7.0 and 8.2 months. Chawla et al. analyzed adrenal SBRT in 30 patients and reported 1-year and 2-year LC rates of 55 and 27%, respectively [27]. Another recently published study included 33 patients and demonstrated a 1-year LC of 58.1% and 2-year LC of 50% for the patients who were still alive [37]. Both Scouarnec et al. and Toesca et al. demonstrated slightly superior local control rates of 92.4–96.5% after 1 year and 80.8–92.6% after 2 years [23, 40]. However, their irradiated tumors showed a median tumor volume of 13.1 and 19 ml, while the median tumor volume in our study was considerably higher with 42 ml for the GTV and 96 ml for the PTV. The larger tumor volumes may have comprised local control in our study. Indeed, Zhao et al. reported that a larger GTV volume significantly correlated with inferior LC following adrenal SBRT [41].

Similar to pulmonary and hepatic SBRT, a clear dose-response relationship is also expected for the treatment of adrenal gland metastases [17, 42]. Chance et al. recently reported no local failures following adrenal SBRT with a BED_10_ > 100 Gy [26]. In line with these results, another analysis demonstrated that adrenal SBRT with a BED_10_ ≥ 85.5 Gy correlated with superior LC [41]. Notably, the patients in the study by Scouarnec et al. were treated with higher total doses in BED_10_ (median 112.5 Gy) compared to the median BED_10_ in our analysis (75.0 Gy), which might also have impaired our results [40]. We also detected a non-significant trend for superior LC if a BED_10_ ≥ 75.0 Gy was applied (*p* = 0.101), both patients diagnosed with a local recurrence were treated with a BED_10_ < 67.5 Gy. However, due to the close proximity of the adrenal glands to the stomach, the duodenum and the small bowel and their intrinsic radiosensitivity, higher doses often could not be safely delivered without potentially increasing toxicity. In this cohort, patients were treated with the highest possible doses, but organ-specific doses constraints regularly caused dose restrictions. This fact is illustrated by the wide range of calculated conformity indices in this study (median CI of 0.5 (range 0.4.-0.99), with 50% of the patients requiring reduced target volume coverage to meet adjacent OAR dose constraints.

The adrenal glands have been reported to show extensive respiration induced motion of more than 20 mm in several directions [22, 43]. To account for target movement due to breathing, nearly all patients were treated with a 4-D-CT-based internal target volume (ITV) concept in our study. However, Cusumano et al. recently reported that the motion amplitude acquired during 4-D-CT does not correlate to the magnitude of drifts or the necessary margins during treatment [44]. Hence, passive motion management strategies like the 4-D-CT-based ITV concept might not always be sufficient and might potentially influence local control following adrenal SBRT.

On the contrary, active motion management strategies like gating and tracking, allow for real-time target monitoring during radiotherapy [22, 45]. When applying gating or tracking, the ITV-concept is no longer needed and PTV sizes can be substantially decreased. Haidenberger et al. analyzed robotic radiosurgery using active tumor tracking at the CyberKnife for adrenal SBRT and reported much smaller PTV sizes of in median 48.6 cm^3^ compared to the median PTV size of 95.9 cm^3^ in our study, in which an ITV-based approach was applied [43]. Due to the smaller PTV sizes and hence the larger distance to surrounding radiosensitive OARs, higher doses potentially leading to superior LC could be delivered when active motion management strategies are used [40, 46]. However, as gating and tracking technologies are usually applied in combination with X-ray based image-guidance, the invasive implantation of fiducial markers in the well vascularized adrenal glands becomes necessary. Scouarnec et al. reported about severe side-effects in 10.7% of the patients following fiducial implantation in the adrenal glands including hematomata and pneumothoraces [40].

Although gating and tumor tracking with robotic radiosurgery enables real-time monitoring of target motion, daily position and movement of the surrounding OARs in the abdomen is not taken into account. Substantial displacements and volume changes for the stomach, bowel, and duodenum are known to occur intra- and interfraction during adrenal SBRT [47]. In clinical routine, the applied treatment plan is based on a snapshot of the anatomy on the planning CT scan, which necessitates highly conservative dose restrictions to adjacent tissues. Holy et al. reported two patients who developed gastric and duodenal ulcers following adrenal SBRT with a BED of 72 Gy [48]. Interestingly, the corresponding calculated dose-volume load to the stomach and the small intestine was below the tolerance level of these organs. The authors attribute the high toxicity to a different filling of the stomach and the small intestine, which were not detected by X-ray-based image-guidance. In our patient cohort, we performed daily image-guidance with MV- or kV-CT and postponed the treatment if different fillings of hollow organs were detected. This might be also a reason why adrenal SBRT in our cohort was tolerated well with no acute or late ≥grade III toxicity.

In contrast, MR-guided radiotherapy, which has recently become clinically available, offers superior soft-tissue contrast compared to X-ray-based techniques, gated dose delivery as well as real-time plan adaptation [49, 50]. Daily interfractional changes in anatomy can immediately be visualized, and the treatment plan can be adjusted accordingly. Palacios et al. reported about respiration-gated, MR-guided SBRT of adrenal metastases in 17 patients, and underlined the high potential of real-time reoptimization of treatment plans to significantly improve target coverage and sparing of OARs [47]. Furthermore, a phase 1 trial of real-time adaptive MR-guided radiation therapy in the treatment of oligometastatic malignancies of the abdomen enrolled 2 patients with adrenal metastases and concluded that real-time adaptive MR-guided radiotherapy enabled safer delivery of SBRT and allowed doses escalation and/or simultaneous OAR sparing when the anatomy-of-the-day was favorable [51]. Based on the results of this study, we recently implemented respiration-gated MR-guided SBRT of adrenal metastases in our department, leading to the safe application of substantially higher total doses with the aim to further increase local control combined with even lower toxicity.

Limitations to this study were mainly caused by the retrospective nature of this analysis. Patient numbers were rather low, but similar to other studies, as adrenal SBRT is still not offered to many patients [3, 21, 22, 24, 36, 43, 45]. Furthermore, for increasing strength and homogeneity of our study, we only focused our analysis on truly oligometastatic or oligoprogressive patients and excluded all patients who were treated with palliative intent or dose. Median post-treatment follow- up time of 9.8 months makes it challenging to assess long-term LC, PFS, OS, and potentially late toxicity. This was a result of 18 patients (64.3%) having died during follow-up, which highly reduced the analyzed timespan. Nevertheless, most of the patients died due to distant progression which emphasizes that patient selection for adrenal SBRT as well as administration of systemic therapy, both are crucial.

SBRT for adrenal metastases resulted in promising local control with only mild toxicity. Based on our study and our data, a dose-response relationship also seems to exist for adrenal SBRTs. However, we could also show that the application of sufficiently high doses is often limited by the close proximity of OARs and further dose escalations strategies and techniques (e.g. gating, tracking, adaptive radiotherapy) should be investigated in future.

## Data Availability

The data used in this analysis is from publications available in the public domain.

## Abbreviations

BED: Biological effective dose
CI: Conformity index
CR: Complete response
CT: Computed tomography
CTV: Clinical target volume
FDG-PET: Fluoro-deoxy-glucose positron emission tomography
FFF: Flattening filter free
GTV: Gross tumor volume
HI: Homogeneity index
IMRT: Intensity-modulated radiotherapy
ITV: Internal target volume
LC: Local control
MRI: Magnetic resonance imaging
OARs: Organs at risk
OS: Overall survival
PD: Progressive disease
PFS: Progression free survival
PR: Partial response
PTV: Planning target volume
SBRT: Stereotactic body radiotherapy
SD: Stable disease
VMAT: Volumetric modulated arc therapy
